# Immune Checkpoint Inhibitor-Induced Subacute Myocarditis

**DOI:** 10.7759/cureus.91464

**Published:** 2025-09-02

**Authors:** Taysir Al Janabi, Milan Patel, Sohaib Roomi, Abhinav Hoskote

**Affiliations:** 1 Internal Medicine, WellSpan York Hospital, York, USA; 2 Internal Medicine, Drexel University College of Medicine, Philadelphia, USA; 3 Heart and Vascular, WellSpan York Hospital, York, USA

**Keywords:** cardiotoxicity, ici, immune checkpoint inhibitors, immune-related adverse events, myocarditis

## Abstract

Immune checkpoint inhibitors (ICIs) are a novel class of drugs that direct the immune system to destroy cancer cells, thereby significantly improving patient survival rates. ICI-induced myocarditis, though uncommon, is the most severe side effect associated with these treatments because of its high mortality rate.

We report the case of an 81-year-old man with a medical history significant for non-small cell lung cancer who had previously undergone chemoradiotherapy and was receiving the ICI durvalumab. He presented with progressive shortness of breath, and his clinical picture was complicated by pneumonia. His laboratory workup showed evidence of acute kidney injury, elevated liver enzymes, and volume overload. His echocardiogram showed evidence of systolic dysfunction. Cardiac magnetic resonance imaging (MRI) confirmed the diagnosis of subacute myocarditis, likely caused by the ICI.

The main presenting symptoms of ICI-induced subacute myocarditis are arrhythmia and heart failure based on the time interval from the initiation of ICI treatment to the onset of symptoms. Our case could have easily been misdiagnosed as a type 2 myocardial infarction because of the patient’s pneumonia. Cardiac MRI was the diagnostic tool for the diagnosis of ICI-induced subacute myocarditis in our case.

Myocarditis should be considered in patients receiving ICIs, particularly those undergoing combination therapy, because it can be misdiagnosed as a type 2 myocardial infarction.

## Introduction

Myocarditis is an uncommon but potentially fatal adverse effect of immune checkpoint inhibitors (ICIs). ICIs are a novel class of drugs that direct the immune system to recognize and destroy cancer cells. They are widely used because of their ability to bring about notable improvements in terms of clinical response, tumor reduction, and survival [1]. There are three main classes of ICIs, each with a distinct mechanism of action: those targeting programmed cell death protein 1 (PD-1), those targeting programmed death ligand 1 (PD-L1), and those targeting cytotoxic T-lymphocyte-associated protein-4 (CTLA-4) [2]. For example, durvalumab is a PD-L1 inhibitor that was approved in 2017 for non-small cell lung cancer (NSCLC) [3].

During the initial regulatory approval of ICIs in 2014, immune-related adverse effects (irAEs) were reported to occur in approximately 80%-90% of cases and to involve various organ systems, including the nervous, endocrine, gastrointestinal, respiratory, and renal systems. A few cases of ICI-induced myocarditis were reported after ICIs received approval [4].

ICI-induced myocarditis is infrequent, but it is the most serious irAE of ICIs, with a mortality rate of up to 50% [5]. Its incidence is reported to be 0.3%-1.4% of all ICI-treated cases, with most of the data being based on post-marketing surveillance [6]. The condition is commonly associated with anti-PD-L1, and the risk is highest among those receiving combination immunotherapy, accounting for 25% of combination therapy deaths, with a median time of onset of 30 days from the initiation of an ICI [6,7]. Additional risk factors for ICI-induced myocarditis include diabetes mellitus, hypertension, and smoking [8]. Histopathologically, ICI-induced myocarditis is mediated by T-cell and macrophage infiltration with subsequent cardiomyocyte death, though these findings are not specific to ICIs [1,5]. Clinically, myocarditis can present as a spectrum ranging from a mild asymptomatic rise in cardiac biomarkers (isolated troponin elevation) to a severe form resulting in cardiogenic shock or life-threatening arrhythmia, such as advanced atrioventricular block or ventricular tachycardia [1,9]. The fact that nearly all documented cases of ICI-induced myocarditis have been severe may explain why this illness has a high mortality rate [9]. Additionally, the mild form of ICI-induced myocarditis may be reported as a type 2 myocardial infarction (demand ischemia) because of the modest elevation in patients’ cardiac biomarkers [6].

Here, we present a case of ICI-induced myocarditis that is notable for having a subacute course.

## Case presentation

An 81-year-old male presented with a medical history significant for coronary artery disease status post (s/p) coronary artery bypass graft, percutaneous coronary intervention (PCI), atrial fibrillation s/p watchman without anticoagulation because of gastrointestinal bleeding, left internal carotid artery stenosis, hypertension, type 2 diabetes mellitus, and non-small cell lung cancer s/p chemoradiotherapy, and he was currently on durvalumab. The patient came to the hospital with complaints of progressive shortness of breath and fatigue over a period of weeks. Prior to visiting the hospital, he contracted a viral upper respiratory infection that was complicated by pneumonia, and for which he received antibiotics that did not improve his symptoms. In the emergency department, his vitals were significant for a temperature of 97.3°F, SpO2 at 86%, requiring 4 L oxygen through nasal cannula, and blood pressure of 116/81 mmHg. His physical examination was notable for elevated jugular venous pressure, bilateral lung rales, and 2+ bilateral leg edema.

**Figure 1:**
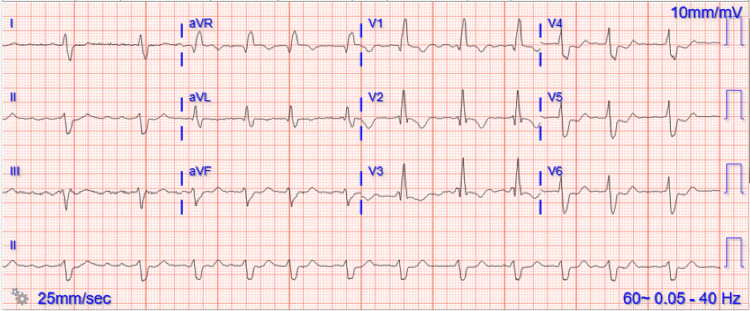
Electrocardiogram upon presentation showing atrial fibrillation.

**Figure 2:**
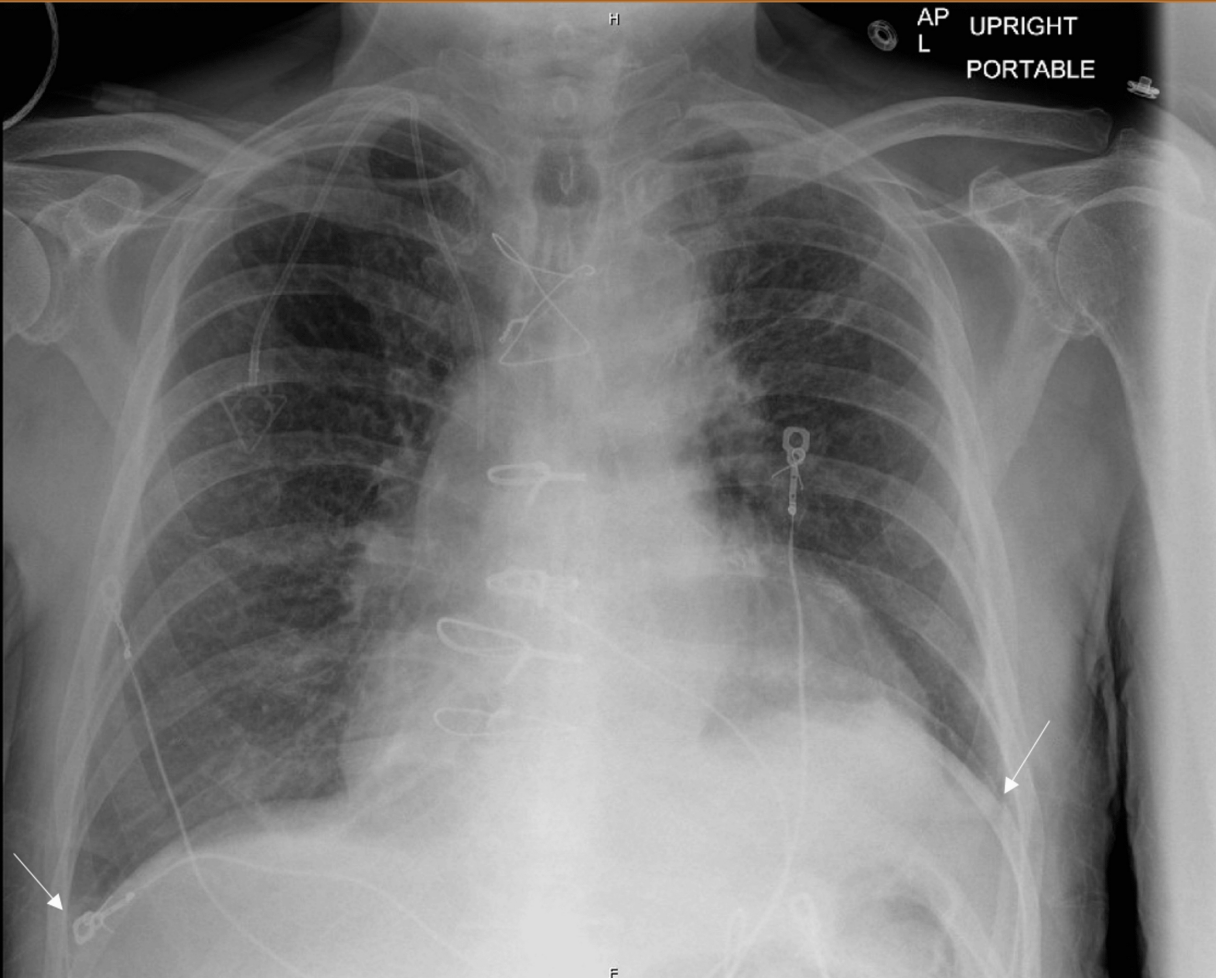
Chest X-ray showing cardiomegaly and pulmonary edema.

Comprehensive laboratory studies revealed anemia, thrombocytopenia, acute kidney injury, elevated liver enzymes, and evidence of volume overload (Table 1). The patient’s respiratory viral panel was negative, and an electrocardiogram (EKG) showed irregular rhythm consistent with atrial fibrillation (Figure 1). His chest X-ray showed cardiomegaly with evidence of pulmonary edema (Figure 2). His chest CT pulmonary embolism protocol did not show evidence of pulmonary embolism, but it did show moderate bilateral pleural effusions. His transthoracic echocardiogram (TTE) showed a moderately dilated left ventricle (LV) with severely reduced systolic function, an ejection fraction (EF) of 20%-25%, and diffuse hypokinesis. A TTE four years previously had shown concentric left ventricular hypertrophy (LVH) and inferior wall hypokinesis along with a low normal function EF of 50%.

**Table 1:** Laboratory studies.

Test	Results	Reference range
Hemoglobin	10.8 g/dl	13.0–17.3 g/dl
Platelets	138 K/mcL	140–400 K/mcL
Creatinine	1.62 mg/dl	0.70–1.30 mg/dl
Aspartate aminotransferase	61 IU/L	13–39 IU/L
Alanine aminotransferase	56 IU/L	7–52 IU/L
B-type natriuretic peptide	2,047 pg/mL	≤100 pg/mL
High sensitivity troponin	814 ng/L	ng/L

**Figure 3:**
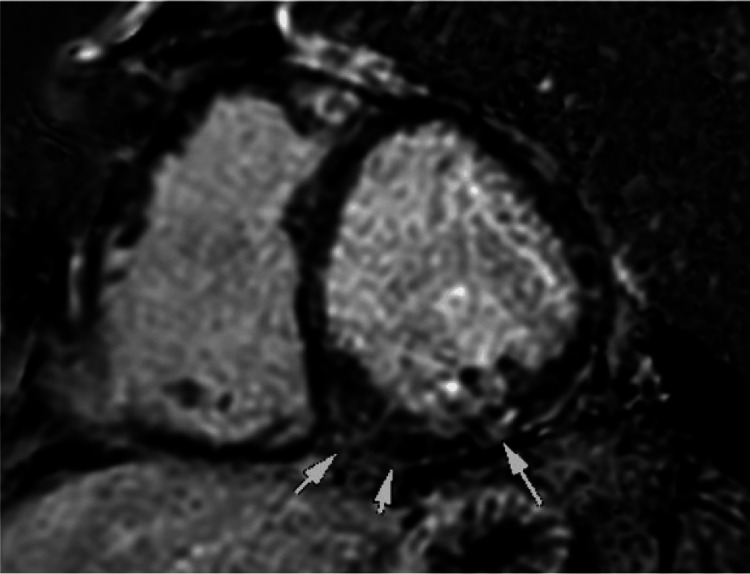
Cardiac MRI showing dense patchy enhancement was noted within the mid inferoseptum near the insertion point of the RV along with scattered areas of subepicardial enhancement within the mid inferior and mid inferolateral walls. MRI: Magnetic resonance imaging, RV: Right ventricle

Based on the echocardiographic findings, cardiology was consulted, and the recommended PET/CT myocardial perfusion imaging showed a large, predominantly reversible filling perfusion defect in the anterior, anterolateral, and anteroseptal wall segments, suggestive of ischemia in the left anterior descending (LAD)/left internal mammary artery (LIMA) region. Cardiac magnetic resonance imaging (CMR) revealed a severely reduced systolic function of the LV and right ventricle (RV), with an estimated ejection fraction (EF) of 16% and 17%, respectively. Additionally, global dysfunction with regional variation was observed: the interventricular septum, anterior wall, and lateral walls were akinetic, while the inferior wall was severely hypokinetic. Moreover, a dense, patchy enhancement was noted within the mid inferoseptum near the insertion point of the RV, along with scattered areas of subepicardial enhancement within the mid inferior and mid inferolateral walls (Figure 3).

The CMR findings were concerning for non-ischemic processes and subacute myocarditis. The durvalumab was immediately discontinued, and the patient was started on a tapering weight-based prednisone of 80 mg daily for one week, then tapered by 10 mg weekly for a total course of eight weeks. The patient also started on milrinone infusion for optimization prior to right and left heart catheterization and possible high-risk PCI. Heart catheterization showed severe stenosis, 95%, at the ostium of the left circumflex artery and severe proximal stenosis of the saphenous vein graft (SVG) to the diagonal, both of which were stented. The cardiac index was low, 1.4. The patient was started on dual antiplatelet therapy (DAPT) with aspirin and Brilinta for one year and then continued on acetylsalicylic acid (ASA) for life. As part of the guideline-directed medical therapy (GDMT) for heart failure with reduced ejection fraction (HFrEF), the patient was started on Jardiance. The rest of the GDMT was initiated on an outpatient basis for as long as his blood pressure and heart rate would tolerate it. The patient was discharged with a life vest in view of his low EF. A three-month follow-up visit revealed improvement in the patient’s EF from 20%-25% to 45%-50%. Additionally, there was an improvement in biomarkers, with a decrease in B-type natriuretic peptide (BNP) from 2,047 pg/mL to 435 pg/mL and troponin from 814 ng/L to 38 ng/L. The patient did not require an implantable cardioverter-defibrillator.

## Discussion

ICI-induced myocarditis is a life-threatening condition, with the median onset of symptoms occurring 30 days after the initiation of ICI treatment. However, symptoms may occur within 15 days among patients on combination ICI therapy. Most initial reports of ICI-induced myocarditis are fulminant, with early onset of symptoms, and these results suggest that less severe or subacute ICI-induced myocarditis has been underreported. Fulminant ICI-induced myocarditis classically begins shortly after ICI treatment is initiated. However, late-onset ICI-induced myocarditis has also been reported. An autopsy diagnosis of ICI-induced myocarditis was reported by Nguyen et al., developed 131 weeks after the initiation of an ICI (and 33 weeks after the ICI was discontinued) [6]. The timeline of our patient's presentation is more likely consistent with a subacute presentation, for he received 11 cycles of monthly durvalumab before the onset of symptoms (approximately 44 weeks after the ICI was initiated).

The clinical presentation of ICI-induced myocarditis varies, potentially in relation to the timing of the onset of symptoms. Arrhythmia is the likely presentation of early-onset ICI-induced myocarditis, while heart failure is a common presentation of late-onset myocarditis. Dolladille et al. reported 19 cases of late-onset ICI-induced myocarditis (with a median time to onset of 304 days from the initiation of treatment), and their findings indicated that heart failure was the main presentation in 50% of these cases, compared with 5% in early-onset cases (with a median time to onset of 14 days from the initiation of ICI) [6]. The heart failure presentation may be attributable to LV systolic dysfunction, which is also common in late-onset ICI-induced myocarditis [8]. Our patient's presentation was consistent with the available literature in that HFrEF was the clinical presentation.

Once the diagnosis of ICI-induced myocarditis is established, immunotherapy should be discontinued immediately, and corticosteroid therapy should be initiated. Up to 50% of cases fail to respond to corticosteroids; thus, immunosuppressive therapy should be considered as a second-line treatment [9]. The treatment team decided to start with weight-based prednisone at 1 mg/kg daily with a taper course by 10 mg weekly for a total course of eight weeks. An approach consistent with the American Society of Clinical Oncology (ASCO) clinical practice guidelines [8]. Our patient demonstrated gradual improvement in both clinical and laboratory findings following oral prednisone treatment.

While there is no consensus on the screening, surveillance, prevention, or even treatment of ICI-induced myocarditis, many professional medical societies, including the Society for Immunotherapy of Cancer, the European Society of Medical Oncology, and the European Society of Cardiology, recommend obtaining baseline EKG, troponin, and B-type natriuretic peptide levels prior to initiating ICI treatment, and a baseline echocardiogram is also recommended for high-risk individuals [8]. No baseline cardiac biomarkers were established for our patient.

A main limitation to this case is the concurrent occurrence of ischemic and non-ischemic cardiomyopathy, making it challenging to ascertain the causation of Durvalumab in this patient's presentation. While cardiac MRI is highly suggestive, endomyocardial biopsy is the diagnostic gold standard [10]."

## Conclusions

Subacute myocarditis, though rare, poses a serious threat to patients undergoing ICI therapy, especially those receiving combination regimens. The fact that its clinical course often leads to misdiagnosis as a type 2 myocardial infarction underscores the importance of heightened clinical awareness and accurate diagnostic strategies to ensure timely and appropriate management.
